# Middle East Respiratory Syndrome Coronavirus Infection Dynamics and Antibody Responses among Clinically Diverse Patients, Saudi Arabia

**DOI:** 10.3201/eid2504.181595

**Published:** 2019-04

**Authors:** Hail M. Al-Abdely, Claire M. Midgley, Abdulrahim M. Alkhamis, Glen R. Abedi, Xiaoyan Lu, Alison M. Binder, Khalid H. Alanazi, Azaibi Tamin, Weam M. Banjar, Sandra Lester, Osman Abdalla, Rebecca M. Dahl, Mutaz Mohammed, Suvang Trivedi, Homoud S. Algarni, Senthilkumar K. Sakthivel, Abdullah Algwizani, Fahad Bafaqeeh, Abdullah Alzahrani, Ali Abraheem Alsharef, Raafat F. Alhakeem, Hani A. Aziz Jokhdar, Sameeh S. Ghazal, Natalie J. Thornburg, Dean D. Erdman, Abdullah M. Assiri, John T. Watson, Susan I. Gerber

**Affiliations:** Ministry of Health, Riyadh, Saudi Arabia (H.M. Al-Abdely, A.M. Alkhamis, K.H. Alanazi, W.M. Banjar, O. Abdalla, M. Mohammed, H.S. Algarni, A. Alzahrani, A.A. Alsharef, R.F. Alhakeem, H.A.A. Jokhdar, A.M. Assiri);; Centers for Disease Control and Prevention, Atlanta, Georgia, USA (C.M. Midgley, G.R. Abedi, X. Lu, A.M. Binder, A. Tamin, S. Lester, R.M. Dahl, S.K. Sakthivel, N.J. Thornburg, D.D. Erdman, J.T. Watson, S.I. Gerber);; Princess Nourah Bint Abdulrahman University, Riyadh (W.M. Banjar);; Prince Mohammed Bin Abdulaziz Hospital, Riyadh (A. Algwizani, F. Bafaqeeh, S.S. Ghazal)

**Keywords:** Middle East respiratory syndrome, coronavirus infections, diabetes mellitus, kinetics, viral load, antibody response, asymptomatic infections, mortality, viruses, respiratory infections, MERS, MERS-CoV

## Abstract

Middle East respiratory syndrome coronavirus (MERS-CoV) shedding and antibody responses are not fully understood, particularly in relation to underlying medical conditions, clinical manifestations, and mortality. We enrolled MERS-CoV–positive patients at a hospital in Saudi Arabia and periodically collected specimens from multiple sites for real-time reverse transcription PCR and serologic testing. We conducted interviews and chart abstractions to collect clinical, epidemiologic, and laboratory information. We found that diabetes mellitus among survivors was associated with prolonged MERS-CoV RNA detection in the respiratory tract. Among case-patients who died, development of robust neutralizing serum antibody responses during the second and third week of illness was not sufficient for patient recovery or virus clearance. Fever and cough among mildly ill patients typically aligned with RNA detection in the upper respiratory tract; RNA levels peaked during the first week of illness. These findings should be considered in the development of infection control policies, vaccines, and antibody therapeutics.

Infection with Middle East respiratory syndrome (MERS) coronavirus (MERS-CoV) results in a wide range of clinical manifestations, from mild or asymptomatic illness to severe respiratory failure (*1*–*8*); infection has a reported mortality rate of 35% (*9*). Most MERS cases have been reported in older adults with underlying medical conditions (*4*,*7*). Asymptomatic or mild infections are typically reported in younger, healthy adults, including healthcare personnel (*2*,*4*). MERS-CoV transmission is commonly associated with exposure to symptomatic patients in healthcare (*1*,*2*,*10*,*11*) or household (*12*) settings or with direct exposure to dromedary camels (*13*).

Infection prevention and control guidance for MERS-CoV in humans is partially based on severe acute respiratory syndrome (SARS) coronavirus infection dynamics (*14*,*15*); MERS-specific recommendations are incomplete. Investigations of virus shedding in MERS patients have demonstrated that MERS-CoV RNA can be detected in the respiratory tract for >1 month from illness onset (*16*,*17*); lower respiratory tract (LRT) specimens have higher (*18*–*23*) and often more prolonged RNA levels (*17*,*18*) than upper respiratory tract (URT) specimens; more severely ill patients typically have higher (*18*,*21*) and more prolonged (*18*) RNA levels; and MERS-CoV RNA is detected in the blood (*17*,*22*,*24*), serum (*18*,*19*,*24*), plasma (*22*,*25*,*26*), stool (*19*,*23*,*27*), and urine (*17*,*19*,*23*) of some patients. However, important knowledge gaps remain, particularly regarding shedding in association with clinical manifestations and host factors (*4*).

Serologic responses among MERS patients are incompletely understood; such data are critical for the development of vaccines, antibody therapeutics, and diagnostics. Investigations of MERS survivors have demonstrated that antibody titers are higher and longer-lived in more severely ill patients than in mildly ill patients (*28*), some of whom do not develop a detectable response (*28*,*29*). Antibodies are usually detected by day 21 after illness onset (*30*,*31*) and can persist for >34 months after infection (*32*). Data on case-patients who died, however, are limited (*19*,*25*,*29*).

To address gaps in viral and antibody kinetics, we longitudinally assessed 33 hospitalized MERS-CoV–infected patients. Our aim was to characterize MERS-CoV infection dynamics and antibody responses in relation to outcome, clinical manifestations, underlying medical conditions, and preillness exposures.

## Methods

### Patient Enrollment

The study population was drawn from a MERS referral hospital in Riyadh, Saudi Arabia. All patients testing positive for MERS-CoV locally by real-time reverse transcription PCR (rRT-PCR) assay and admitted to this hospital during August 1, 2015–August 31, 2016, were eligible for participation. All enrolled patients provided informed written consent.

### Data Collection

We reviewed epidemiologic interviews conducted at the time of case identification to include patient demographics, symptom history, and relevant exposures during the 2 weeks before onset. After patient death or discharge, we performed comprehensive medical chart reviews to collect medical history; symptoms before hospitalization; and daily information regarding symptoms during hospitalization, clinical course, treatments, medications, patient vital signs, diagnostic tests, and clinical outcome.

To assess MERS-CoV infection status, we retrospectively reviewed 3 data sources (as available) containing information on clinical diagnostic testing: 1) rRT-PCR request forms submitted to a regional testing facility; 2) hospital copies of corresponding results; and 3) if the hospital’s clinical series was incomplete, rRT-PCR results from the Health Electronic Surveillance Network (*33*), a national platform for reporting notifiable diseases in Saudi Arabia. MERS-CoV clinical diagnostic testing had been performed on URT or LRT specimens typically collected every other day throughout hospitalization. Healthcare personnel collected LRT specimens from intubated patients and URT specimens otherwise. MERS-CoV results were positive, probable, or negative and, if available, cycle threshold (C_t_) values for MERS-CoV upstream of the envelope E (upE) or open reading frame (ORF) 1a (*34*); a probable finding indicated that only 1 of these 2 targets was detected.

### Laboratory Investigation 

In addition to retrospectively reviewing clinical MERS-CoV test results, we periodically collected specimens throughout hospitalization for molecular and serologic testing at the US Centers for Disease Control and Prevention (CDC). Specimens were collected from respiratory and nonrespiratory sites, frozen at <–70°C, and shipped on dry ice. Available specimens were URT (nasopharyngeal, oropharyngeal swab, or combined), LRT (sputum or tracheal aspirate), whole blood, serum, stool, and urine. Specimens were collected during days 1–42 postenrollment and additionally at 1 year for serum.

#### Molecular Assays

Specimens were processed and screened by upE and N2 rRT-PCR. Specimens positive by only 1 RT-PCR were confirmed by N3 assay as previously described (*35*). MERS-CoV isolation was performed as previously described (*36*). We attempted full genome sequencing, as previously described (*36*), on the earliest available respiratory specimen (or serum, if not available) for each patient.

#### Serologic Assays

Serum specimens with sufficient volume were tested using 4 CDC serologic assays: 1) microneutralization (MN) assay (*37*); 2) spike (S)–specific pseudoparticle neutralization assay (VSV-MERS-S); 3) S ELISA (Ig-specific) (*38*); and 4) nucleocapsid (N) ELISA (Ig-specific) (*37*,*38*). Additional description is available in Appendix 1.

### Data Analysis

#### Definitions

We defined illness onset as the first day of reported symptoms consistent with MERS; for asymptomatic patients identified through routine contact investigations, we used the date of the first positive MERS-CoV test. We analyzed data relative to the date of illness onset (day 0). Patients were classified as having diabetes mellitus (DM) if there was a documented medical history of DM. Patients with multiple periods of hyperglycemia during hospitalization (random glucose readings >200 mg/dL), but with no documented medical history of DM, were considered as possible DM status. 

Cardiac disease included congestive heart failure, coronary artery disease, or a history of myocardial infarction; reports of isolated hypertension were not included. Pulmonary disease included chronic obstructive pulmonary disease, asthma or reactive airway disease, or use of supplemental oxygen at home. Renal disease included reports of chronic kidney disease. Secondary exposure was defined as contact with MERS-CoV–infected persons in the 2 weeks before illness onset. Primary exposure was defined as either reported direct camel contact or no known contact with MERS-CoV–infected persons.

#### Illness Severity

We retrospectively categorized patients into 3 groups on the basis of the need for supplemental oxygen, ventilation, and clinical outcome. Group 1 (G1) received room air throughout hospitalization; group 2 (G2) required ventilator support (mechanical or nonmechanical) and survived; and group 3 (G3) required ventilator support and died.

#### MERS-CoV Detection Period

To analyze duration of detectable MERS-CoV among survivors, we assessed the number of days from illness onset to negativity in clinical respiratory specimens tested at the regional testing facility, based on reports from the hospital or the Health Electronic Surveillance Network. We defined the day of MERS-CoV negativity as the first of >2 consecutive negative tests before discharge. These variables were based on either URT or LRT specimens. Because mildly ill patients did not provide LRT specimens, we only assessed detection in URT specimens when comparing severity groups.

#### Prolonged MERS-CoV Detection

To assess prolonged MERS-CoV detection, we expressed time to negativity as a binary variable: patients with time to negativity <11 versus >11 days. We chose this cutoff to reflect the median time to negativity among survivors. Given the low numbers of patients in our cohort, we also assessed 2 additional cut-offs for prolonged shedding to strengthen statistically significant findings: <14 versus >14 days and <21 versus >21 days.

#### Viral Load

To approximate viral load in clinical results, we assessed MERS-CoV upE rRT-PCR C_t_ values determined at the regional testing facility. We used C_t_ values from LRT specimens to assess mechanically ventilated patients. We were able to identify the minimum C_t_ value (or peak RNA level) in a subset of patients. For specimens submitted to CDC, we estimated viral load on the basis of the upE C_t_ value (or N2 C_t_ value if upE testing was negative and N3 was positive).

#### Antibody Responses

We compared the proportion of serum specimens with detectable antibody responses between survivors and patients who died. We assessed specimens collected <14, <21, and <28 days after illness onset; during 28–56 days after onset; and then at 1 year.

#### Statistical Analyses

We summarized patient characteristics by illness severity and, among survivors, by time to MERS-CoV negativity. We used Fisher exact, Kruskall–Wallis, or log rank tests to compare groups and exact logistic regression for multivariable analysis. We compared antibody titers with estimated viral load in different specimen types by using the Spearman test for correlation. All data were analyzed using Microsoft Excel 2016 (https://products.office.com) and SAS version 9.4 (https://www.sas.com).

## Results

### Cohort Description

**Figure 1:**
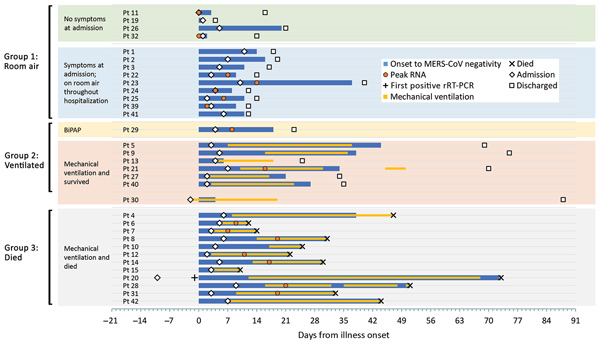
Timeline of clinical course and MERS-CoV detection, by patient, Saudi Arabia, August 1, 2015–August 31, 2016. Findings are presented by time since illness onset (day 0). Patients are grouped by illness severity and outcome. For each patient, day of admission, discharge or death, period of mechanical ventilation (if applicable), and MERS-CoV detection are depicted. For a subset of patients with sufficient data, the peak RNA level (or the minimum upstream of the envelope cycle threshold value) is depicted. Peak RNA was based on upper respiratory tract specimen results among group 1 patients and Pt 29, and lower respiratory tract specimen results in group 2 and group 3 patients. The date of death is shown for group 3 patients. Pt 11 and Pt 32 did not report any symptoms throughout their hospitalization. Pt 30 was hospitalized and mechanically ventilated before MERS onset because of a road traffic accident; this patient was excluded from severity and clinical course analyses. Pt 23 has been described previously (*36*). The first positive MERS-CoV rRT-PCR for Pt 20 was collected 1 day before symptom onset. BiPAP, bilevel positive airway pressure; CoV, coronavirus; MERS, Middle East respiratory syndrome; Pt, patient; rRT-PCR, real-time reverse transcription PCR.

During August 1, 2015–August 31, 2016, a total of 33 MERS-CoV–infected patients were enrolled. Among these, 4 were classified as asymptomatic on admission, and 9 reported symptoms but remained on room air during hospitalization (Figure 1; Appendix 1 Figure 1; Appendix 2 Table 1); 10 of these 13 patients were identified through contact tracing (5 were healthcare personnel) and were hospitalized to ensure isolation. Twenty patients required ventilator support (1 bilevel positive airway pressure [BiPAP] and 19 mechanical ventilation), 12 of whom died. We grouped 13 patients into G1, 7 into G2, and 12 into G3; 1 patient (patient [Pt] 30) was initially hospitalized and intubated after a road traffic accident, before MERS onset, and was excluded from analyses regarding severity and clinical course resulting from MERS-CoV infection.

Patient ages ranged from 26 to 78 years, and 63% were male (Appendix 2 Table 1). Twenty-three (70%) patients had >1 underlying medical condition, 19 of whom had documented DM; an additional 3 patients were considered of possible DM status because they exhibited multiple periods of hyperglycemia (random glucose readings >395 mg/dL) but had no documented history of DM. Death was associated with older age (p<0.001), DM (p = 0.001), hypertension (p<0.001), cardiac disease (p = 0.001), or renal disease (p = 0.001) (Table 1). Among survivors, ventilator support was associated with DM (p = 0.047), older age (p = 0.047), or preillness primary exposure (p = 0.046) (Table 1). Among the 12 patients with a primary exposure, 8 had DM (Appendix 2 Table 1).

**Table 1:** Characteristics of MERS-CoV patients, by clinical severity outcome, Saudi Arabia, August 1, 2015–August 31, 2016*

Characteristic	No. (%) patients							p value†	
	Group 1, n = 13		Group 2, n = 7		Group 3, n = 12			Mortality, G1 and G2 vs. G3	Ventilation support, G1 vs. G2
Demographics									
Sex									
M	7 (54)		6 (86)		7 (58)			0.724	0.329
F	6 (46)		1 (14)		5 (42)				
Nationality									
Saudi	5 (38)		6 (86)		10 (83)			0.139	0.070
Non-Saudi	8 (62)		1 (14)		2 (17)				
Age group, y									
25–44	10 (77)		4 (57)		0			<0.001	0.548
45–64	3 (23)		2 (29)		7 (58)				
>65	0		1 (14)		5 (42)				
Median age (range), y	33 (26–62)		41 (30–73)		62 (55–78)			<0.001	0.047
Underlying conditions									
None reported	10 (77)		0		0			0.004	0.003
Any reported underlying condition	3 (23)		7 (100)		11 (92)				
Unknown underlying condition	0		0		1 (8)				
DM‡	3 (23)		4 (80)		11 (100)			0.001	0.047
DM and possible DM§	3 (23)		6 (86)		12 (100)			0.002	0.047
Hypertension	1 (8)		2 (29)		11 (100)			<0.001	0.270
Cardiac disease¶	0		0		6 (55)			0.001	NA
Pulmonary disease#	0		2 (29)		2 (18)			0.602	0.111
On oxygen at home**	0		1 (14)		1 (9)			1.000	0.350
Renal disease††	0		0		3 (27)			0.001	NA
Cardiac, pulmonary, or renal disease	0		2 (29)		9 (82)			<0.001	0.111
History of cerebrovascular accident	1 (8)		0		3 (27)			0.318	1.000
Cancer in previous 12 mo	0		0		1 (9)			0.355	NA
Possible preillness exposure									
Secondary,‡‡ healthcare personnel	5 (38)		0		0			NA	NA
Secondary, household contact	4 (31)		1 (14)		1 (8)			NA	NA
Secondary, hospital visitor	2 (15)		1 (14)		2 (17)			NA	NA
Secondary, hospital inpatient	0		0		3 (25)			NA	NA
Any secondary exposure	11 (85)		2 (29)		6 (50)			0.249	0.022
Direct camel contact	0		0		2 (17)				
Multiple possible exposures	0		1 (14)		0				
No recognized risks§§	2 (15)		4 (57)		4 (33)				
Primary vs. secondary exposure¶¶									
Primary##	2 (15)		4 (67)		6 (50)			0.452	0.046
Secondary	11 (85)		2 (33)		6 (50)				
Symptoms before admission									
Absence of symptoms	4 (31)		0		0			NA	NA
Any reported symptom	9 (69)		7 (100)		12 (100)			NA	NA
Fever	8 (62)		6 (86)		11 (92)			0.212	0.354
Cough	7 (54)		6 (86)		10 (83)			0.422	0.329
Dyspnea	1 (8)		7 (100)		11 (92)			0.008	<0.001
Vomiting	4 (31)		2 (29)		2 (17)			0.676	1.000
Diarrhea	2 (15)		1 (14)		4 (33)			0.379	1.000
Sore throat	2 (15)		1 (14)		1 (8)			1.000	1.000
Rhinorrhea	1 (8)		1 (14)		1 (8)			1.000	1.000

*Group 1, on room air; group 2, ventilated but survived; group 3, died. CoV, coronavirus; DM, diabetes mellitus; MERS, Middle East respiratory syndrome; NA, not applicable. †p values are for Fisher exact or Kruskall–Wallis tests comparing long-term and short-term. ‡Based on documented medical history of DM; comparison excludes 3 patients with possible DM status; group 1, n = 13; group 2, n = 5; group 3, n = 11. §Includes 18 patients with a documented history of DM and 3 patients with possible DM status who exhibited multiple periods of hyperglycemia (random glucose readings >395 mg/dL) but who had no documented history of DM. ¶Cardiac disease includes congestive heart failure, coronary artery disease, a history of coronary artery bypass, or a history of myocardial infarction. Reports of isolated hypertension were not included. #Pulmonary disease includes chronic obstructive pulmonary disease, asthma or reactive airway disease, or use of supplemental oxygen at home. **Patients who were on supplemental oxygen at home (both patients were on bilevel positive airway pressure) required mechanical ventilation when hospitalized. ††Renal disease includes reports of chronic kidney disease. ‡‡Secondary exposure defined as reported contact with a known MERS case-patient. §§No recognized risks defined as no reported contact with a known MERS case-patient or camel (direct or indirect contact). ¶¶Comparison excludes 1 patient with multiple exposures; group 2, n = 6. ##Primary exposure defined as no reported contact with a known MERS case-patient; includes direct camel contact and patients with no recognized risks.

Clinical course and time to MERS-CoV negativity (in clinical respiratory specimens) is depicted according to date of illness onset (Figure 1; Appendix 2 Table 2). Time to admission (median 4 days) did not differ between groups. Time to MERS-CoV negativity among survivors ranged from day 1 to day 44 after illness onset and was typically longer among G2 than G1 patients. Twelve of 13 patients in G1 were discharged by day 21 after onset; the mildly ill patient who was in the hospital until day 40 after onset (Pt 23) has been described previously (*36*). Duration of hospitalization for G2 patients was 19–70 days, and duration of intubation was 14–31 days. G3 patients died 10–73 days after onset.

### Daily Symptoms

Common symptoms before admission were fever (78%), cough (72%), and dyspnea (59%) (Table 1). Dyspnea before admission was associated with a more severe outcome (p<0.001). Among the 4 patients who reported no symptoms on admission, 2 were mildly symptomatic during hospitalization (Appendix 2 Table 3).

**Figure 2:**
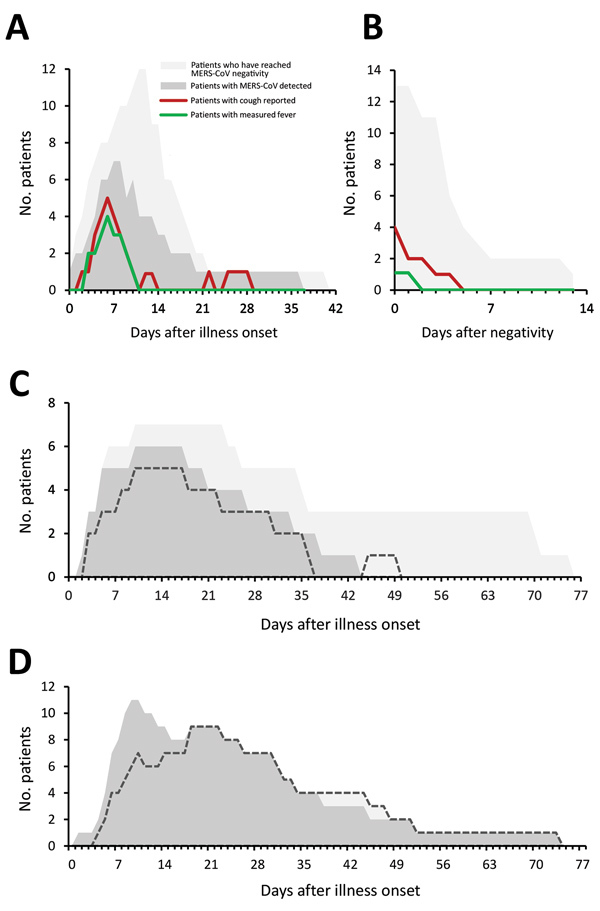
Symptom progression and MERS-CoV detection during hospitalization at a MERS referral hospital, Saudi Arabia, August 1, 2015–August 31, 2016. Each panel depicts the number of patients hospitalized on a given day for each category shown; MERS-CoV detection is based on the clinical diagnostic reports in the upper or lower respiratory tract. A, B) Number of group 1 patients with fever (measured oral temperature >38.0°C or measured axillary temperature >37.5°C) and reported cough during (A) and after (B) the MERS-CoV detection period. C, D) Number of patients intubated (dashed lines) and the number of patients who were positive for MERS-CoV on a given day for group 2 (C) and group 3 (D). MERS, Middle East respiratory syndrome; MERS-CoV, Middle East respiratory syndrome coronavirus. Group 1, on room air; group 2, ventilated but survived; group 3, died.

Among G1 patients, fever and cough were commonly reported, and the proportion of patients with either symptom appeared to align with the proportion who concurrently had detectable MERS-CoV in clinical respiratory specimens (Figure 2, panel A). Cough persisted in 5 G1 patients for <4 days after MERS-CoV negativity (Figure 2, panel B). Chest radiographs of 4 G1 patients were described as abnormal, typically with unilateral findings (Appendix 2 Table 4). Oxygen saturation remained >92% in G1 patients. Among G2 patients, the proportion of patients mechanically ventilated appeared to align with the proportion who had detectable MERS-CoV in the LRT (Figure 2, panel C); only 1 G2 patient (Pt 13, who had underlying pulmonary disease) was MERS-CoV–positive for a short period but required extended mechanical ventilation. Among the 12 G3 patients, 11 were MERS-CoV RNA–positive until death (Figure 2, panel D).

### MERS-CoV RNA in Respiratory Specimens

**Figure 3:**
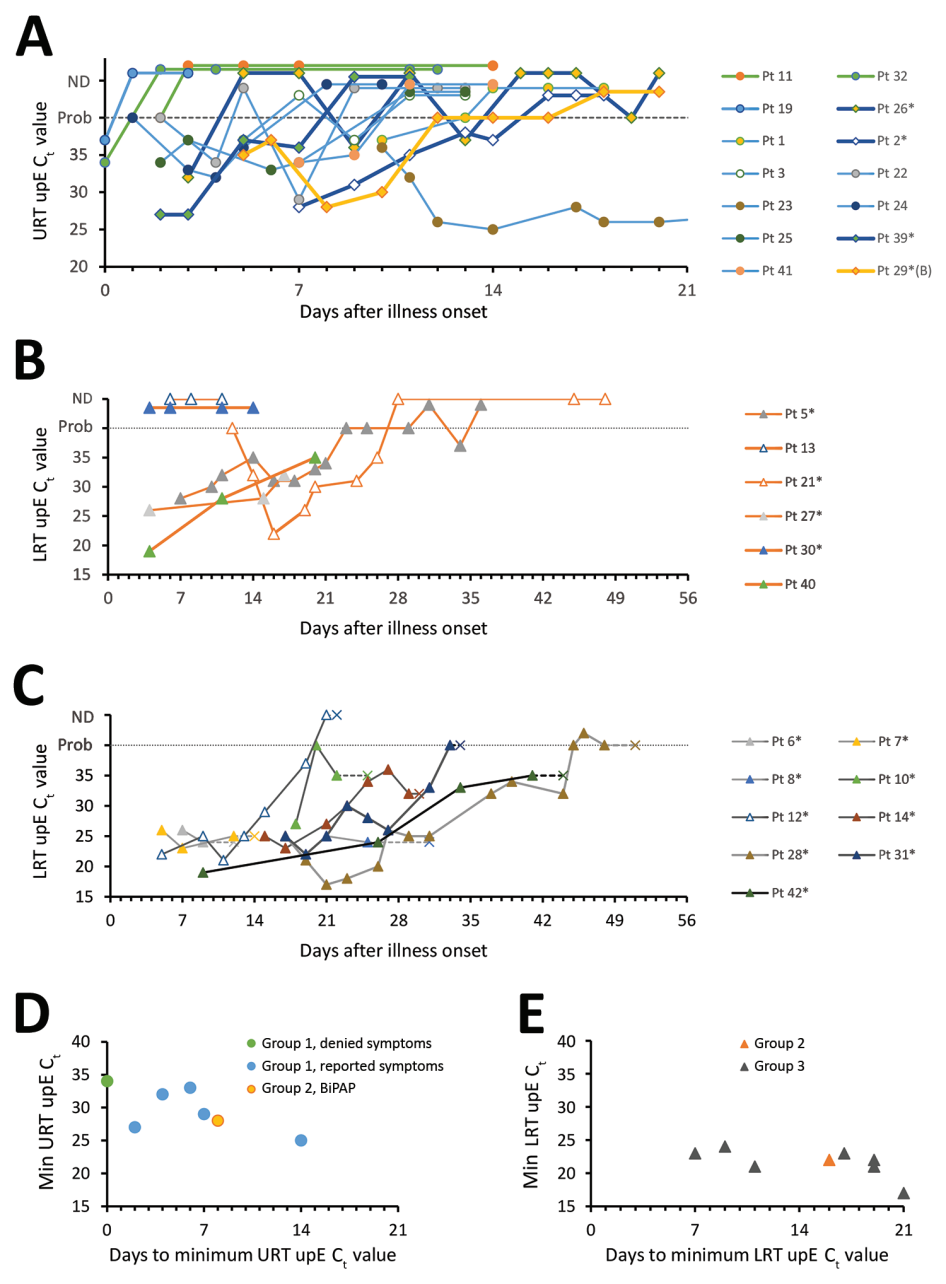
MERS-CoV RNA detection in the respiratory tract, based on clinical diagnostic reports, among MERS-CoV patients, Saudi Arabia, August 1, 2015–August 31, 2016. A–C) UpE real-time reverse transcription PCR C_t_ values of group 1 (A), 2 (B), and 3 (C) patients, by days since illness onset (day 0). Panel A depicts URT specimens, and panels B and C depict LRT specimens collected during MV; Pt 29 (a G2 patient who received BiPAP ventilation) is depicted in panel A because only URT specimens were collected for this patient. The dashed line represents the limit of detection, above which specimens were considered MERS-CoV–negative or not detected. Probable results, meaning that only 1 of 2 real-time reverse transcription PCR assays were positive, are depicted on the dashed line for graphing purposes. Patients with limited C_t_ values or unknown specimen types are not depicted. Patients 11 and 32 did not report any symptoms throughout their illness. Pt 30 is depicted alongside G2 patients. Pt 23 reached negativity 37 days after illness onset, as described previously (*36*). *Indicates patients with a documented history of diabetes mellitus. D, E) Minimum C_t_ values reported, which was determined for a subset of patients with sufficient data. Panel D depicts URT specimen results among group 1 patients and Pt 29; panel E depicts LRT specimen results in group 2 and 3 patients, collected from the LRT during MV. Group 1, on room air; group 2, ventilated but survived; group 3, died. BiPAP, bilevel positive airway pressure; C_t_, cycle threshold; CoV, coronavirus; LRT, lower respiratory tract; MERS, Middle East respiratory syndrome; min, minimum; MV, mechanical ventilation; Pt, patient; URT, upper respiratory tract; upE, upstream of the envelope.

MERS-CoV upE C_t_ values from clinical diagnostic reports are depicted in Figure 3. MERS-CoV RNA levels in the URT of most G1 patients peaked in the first week after onset (Figure 3, panel D). Among patients who died, RNA levels peaked in the LRT during weeks 2 and 3 (Figure 3, panel E), after which RNA levels typically began to decrease (Figure 3, panel C); 4 patients died with negative or probable rRT-PCR results.

We next assessed characteristics of survivors with prolonged MERS-CoV detection periods (on the basis of clinical diagnostic reports of URT specimens) (Tables 2, 3; Appendix 2 Tables 5, 6). Patients who reached negativity >11 days after onset were more likely to have DM than patients who cleared the virus earlier (p = 0.049; when adjusting for severity group, p = 0.061) (Tables 2, 3). This association was also observed in patients who reached negativity >14 days after onset (p = 0.013) and when adjusting for severity group (p = 0.030) (Appendix 2 Table 5). Evidence for this association was stronger when patients with DM and possible DM were combined (Tables 2, 3; Appendix 2 Table 5). No other underlying medical conditions were associated with prolonged detection. Survivors with prolonged detection (>14 or >21 days) were also more likely to require ventilator support (Appendix 2 Table 5), but this was not significant when adjusting for DM.

**Table 2:** Demographic and exposure characteristics of survivors with prolonged MERS-CoV detection, Saudi Arabia, August 1, 2015–August 31, 2016*

Characteristic	Total, N = 19	Days to negativity		p value†
		<11 d, n = 11	>11 d, n = 8	
Demographics				
Sex				
M	13/19 (68)	6/11 (55)	7/8 (88)	0.177
F	6/19 (32)	5/11 (45)	1/8 (12)	
Nationality				
Saudi	10/19 (53)	5/11 (45)	5/8 (63)	0.650
Non-Saudi	9/19 (47)	6/11 (55)	3/8 (37)	
Age group, y				
25–44	14/19 (74)	9/11 (82)	5/8 (63)	0.262
45–64	4/19 (21)	1/11 (9)	3/8 (38)	
>65	1/19 (5)	1/11 (9)	0/8	
Median age, y (range)	36 (26–73)	30 (26–73)	40 (27–62)	0.083
Underlying conditions				
None reported	10/19 (53)	8/11 (73)	2/8 (25)	0.070
Any reported underlying condition	9/19 (47)	3/11 (27)	6/8 (75)	
DM‡	7/18 (39)	2/11 (20)	5/7 (71)	0.049
DM and possible DM§	8/19 (42)	2/11 (20)	6/8 (75)	0.024
Hypertension	3/19 (16)	1/11 (9)	2/8 (25)	0.546
Cardiac disease¶	1/19 (5)	1/11 (9)	0/8	1.000
Pulmonary disease#	1/19 (5)	1/11 (9)	0/8	1.000
On oxygen at home**	1/19 (5)	1/11 (9)	0/8	1.000
Possible preillness exposure				
Secondary,†† healthcare personnel	5/19 (26)	4/11 (36)	1/8 (13)	NA
Secondary, household contact	5/19 (26)	3/11 (27)	2/8 (25)	NA
Secondary, hospital visitor	3/19 (16)	0/11	3/8 (38)	NA
Secondary, hospital inpatient	0/19	0/11	0/8	NA
Any secondary exposure	13/19 (68)	7/11 (64)	6/8 (75)	0.796
Direct camel contact	0/19	0/11	0/8	
Multiple possible exposures	2/19 (11)	1/11 (9)	1/8 (13)	
No recognized risks‡‡	4/19 (21)	3/11 (27)	1/8 (13)	
Primary vs. secondary exposure§§				
Primary¶¶	4/17 (24)	3/10 (30)	1/7 (14)	0.603
Secondary	13/17 (78)	7/10 (70)	6/7 (86)	

*Values are no. (%) patients except as indicated. CoV, coronavirus; DM, diabetes mellitus; MERS, Middle East respiratory syndrome; NA, not applicable. †p values are for Fisher exact or Kruskall–Wallis tests comparing long-term and short-term. ‡Based on documented medical history of DM; comparison excludes 1 patient with possible DM status; total, N = 18; prolonged shedding >11 days, n = 7. §Includes 7 patients with a documented history of DM and 1 patient (of possible DM status) who had no documented history of DM but exhibited multiple periods of hyperglycemia (>200 mg/dL) during hospitalization, with a maximum random glucose reading of 404 mg/dL. ¶Cardiac disease includes congestive heart failure, coronary artery disease, a history of coronary artery bypass, or a history of myocardial infarction. Reports of isolated hypertension were not included. #Pulmonary disease includes chronic obstructive pulmonary disease, asthma or reactive airway disease, or use of supplemental oxygen at home. **Patient who was on supplemental oxygen (bilevel positive airway pressure) at home required mechanical ventilation when hospitalized. ††Secondary exposure defined as reported contact with a known MERS case-patient. ‡‡No recognized risks defined as no reported contact with a known MERS case-patient or camel (direct or indirect contact). §§Comparison excludes 2 patients with multiple exposures; total, N = 17; shed <11 days, n = 10; shed >11 days, n = 7. ¶¶Primary exposure defined as no reported contact with a known MERS case-patient; includes direct camel contact and patients with no recognized risks.

**Table 3:** Clinical features of survivors with prolonged MERS-CoV detection, Saudi Arabia, August 1, 2015–August 31, 2016*

Clinical feature	Total, N = 19	Days to negativity		p value†
		<11 d, n = 11	>11 d, n = 8	
Symptoms before admission‡				
No symptoms	4/18 (21)	3/10 (30)	1/8 (13)	0.603
Fever	13/18 (72)	7/10 (70)	6/8 (75)	1.000
Cough	11/18 (61)	5/10 (50)	6/8 (75)	0.367
Dyspnea	6/18 (33)	2/10 (20)	4/8 (50)	0.321
Vomiting	5/18 (28)	2/10 (20)	3/8 (38)	0.608
Diarrhea	3/18 (17)	0/10	3/8 (38)	0.069
Clinical course‡				
Room air	13/18 (72)	9/10 (90)	4/8 (50)	0.118
Ventilator support§	5/18 (28)	1/10 (10)	4/8 (50)	
Abnormal chest radiograph	9/18 (50)	4/10 (40)	5/8 (63)	0.637
Medications¶				
Ribavirin plus peg-IFNα	2/19 (11)	0/11	2/8 (25)	0.164
Oseltamivir	12/19 (63)	6/11 (55)	6/8 (75)	0.633
Antibiotics	15/19 (79)	8/11 (73)	7/8 (88)	0.603
Parenteral steroids	3/19 (16)	0/11	3/8 (38)	0.058
Group 2 only#	3/5 (60)	0/1	3/4 (75)	0.400
Inhaled steroids	2/19 (11)	0/11	2/8 (25)	0.164
Group 2 only#	2/5 (40)	0/1	2/4 (50)	1.000
Bronchodilators	5/19 (26)	2/11 (18)	3/8 (38)	0.603
Antipyretics	9/19 (47)	6/11 (55)	3/8 (38)	0.650

*Values are no. (%) patients except as indicated. Group 2, ventilated but survived; MERS-CoV, Middle East respiratory syndrome coronavirus. †p values are for Fisher exact or Kruskall–Wallis tests comparing long-term and short-term. ‡Excludes patient no. 30, who was admitted and intubated before onset because of injuries sustained in a road traffic accident (N = 18). §Includes mechanical or nonmechanical (i.e., bi-level positive airway pressure) ventilation. ¶Medication given during MERS-CoV detection period (on the basis of diagnostic testing in respiratory specimens). #Assessing steroid use among G2 patients only.

Based on respiratory specimens submitted to CDC (Appendix 1 Figures 2, 3), full-genome sequences from 13 patients belonged to the NRC-2015 (*39*) clade (or lineage 5 [*40*]) (GenBank accession nos. MG520075 and MG757593–MG757605). Viable MERS-CoV was isolated from 3 of 37 URT specimens; 2 specimens were from a mildly ill patient (Pt 23) collected on days 13 and 15 after onset (described previously [*36*]), and 1 specimen was collected on day 13 from a patient who subsequently died (Pt 8).

### MERS-CoV RNA in Nonrespiratory Specimens

**Figure 4:**
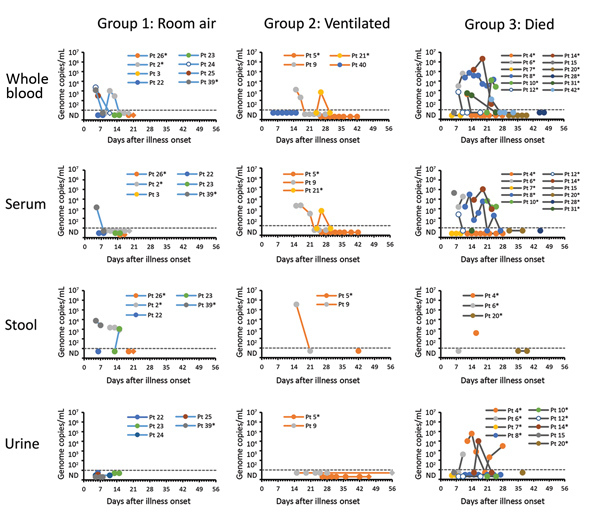
Estimated viral loads in non–respiratory tract specimens collected from hospitalized MERS-CoV patients, Saudi Arabia, August 1, 2015–August 31, 2016, and submitted to the US Centers for Disease Control and Prevention. Specimen types are shown by severity group. Estimated viral loads are based on upstream of the envelope (upE) real-time reverse transcription PCR cycle threshold values, or N2 cycle threshold values if the upE real-time reverse transcription PCR was negative. The dashed line represents the limit of detection, below which specimens were considered MERS-CoV–negative or not detected. Round data points represent specimens collected during the MERS-CoV detection period (defined by clinical results from respiratory specimens). Diamond data points represent specimens collected after the MERS-CoV detection period (defined by clinical results from respiratory specimens); no specimens were positive for MERS-CoV after the detection period. *Patients with a documented history of diabetes mellitus. MERS-CoV, Middle East respiratory syndrome coronavirus; Pt, patient.

CDC received 252 nonrespiratory specimens for MERS-CoV testing, collected from 31 patients <3 months after onset; 7 patients (21 specimens, all MERS-CoV–negative) were excluded because specimens were only collected after the virus had been cleared from the respiratory tract. Fourteen of 24 patients had MERS-CoV RNA detected in whole blood, 9/20 in serum, 5/10 in stool, and 3/16 in urine (Figure 4; Appendix 2 Table 2). In G1, MERS-CoV RNA was detected in the whole blood or serum of 4/8 patients (<2.3 × 10^3^ copies/mL) for <13 days and in the stool of 3/5 patients (<7.5 × 10^3^ copies/mL) for <15 days; only 1 patient with RNA-positive stool had concurrent gastrointestinal symptoms. Specimens were limited in G2, but MERS-CoV RNA was detected in the blood of 2/4 patients (<1.3 × 10^3^ copies/mL) and the stool of 1/2 (3.5 × 10^5^ copies/mL). In G3, high viral loads were detected in the whole blood or serum of 9 patients, reaching as high as 2.1 × 10^6^ copies/mL. All 3 patients with MERS-CoV RNA detected in urine died (<6.0 × 10^4^ copies/mL); 1 patient had chronic kidney disease. We attempted but were unable to isolate live MERS-CoV from 5 stool specimens and 3 urine specimens with elevated MERS-CoV RNA levels.

### Serum Antibody Responses

**Figure 5:**
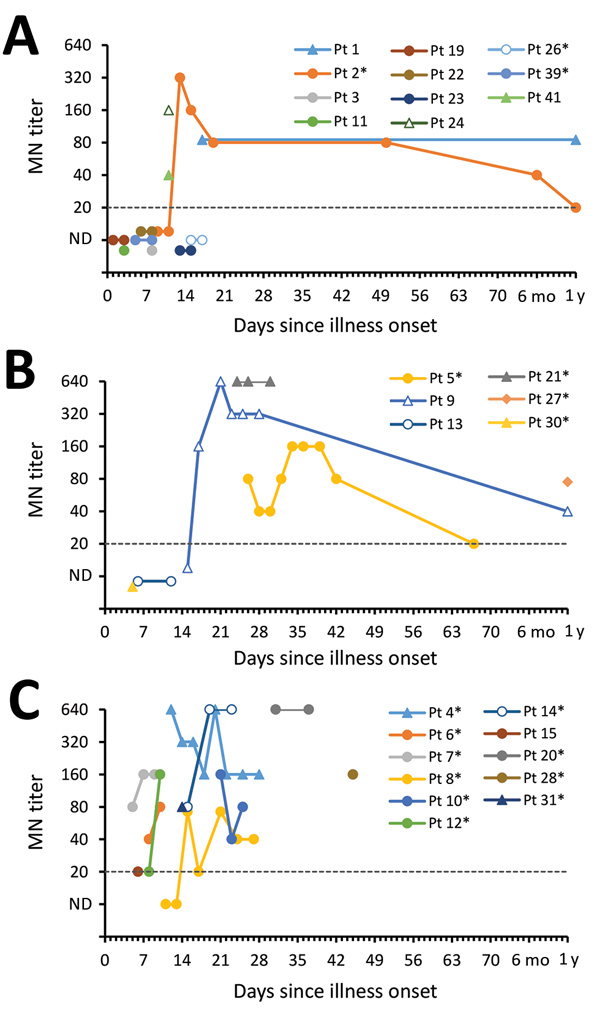
MN antibody titers of serum collected from MERS-CoV patients, by patient, severity group, and days since illness onset (day 0), Saudi Arabia, August 1, 2015–August 31, 2016. A) Group 1 patients; B) group 2 patients; C) group 3 patients. The dashed line represents the limit of detection, below which specimens were considered not to have detectable antibodies. Pt 11 did not report any symptoms throughout their illness. Pt 30 was hospitalized and mechanically ventilated before MERS onset because of a road traffic accident. *Patients with documented history of diabetes mellitus. MERS-CoV, Middle East respiratory syndrome coronavirus; MN, microneutralization assay; ND, antibodies not detected; Pt, patient.

**Figure 6:**
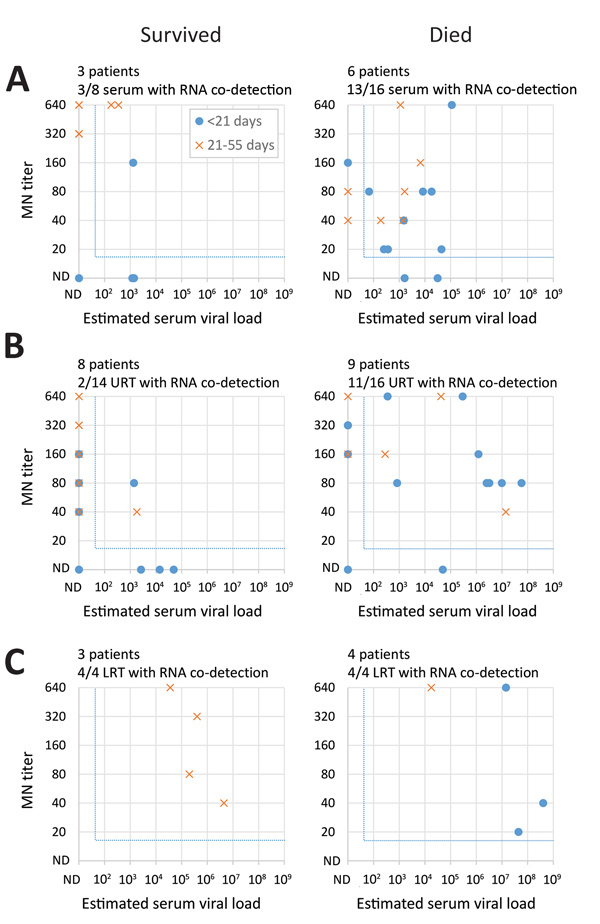
Co-detection of neutralizing serum antibodies with RNA found in serum and the upper and lower respiratory tract among Middle East respiratory syndrome patients, by clinical outcome, Saudi Arabia, August 1, 2015–August 31, 2016. For each patient and specimen, MN titers of serum specimens were compared with estimated viral loads in the same serum specimen (A) or in URT (B) and LRT (C) specimens collected on the same day from the same patient. We defined RNA co-detection as the detection of both RNA and neutralizing antibodies (MN) in the same specimen or in respiratory specimens collected on the same day from a given patient. We only included specimens from patients who were known to develop neutralizing antibodies at any point during or after their illness. For the comparison in serum specimens, we only included specimens from patients who were known to have RNA detected in serum, at any point during their illness. For each panel, the number of patients included are indicated above the panel. The number of specimens with RNA co-detection (indicated by X) among those with detectable antibodies (indicated by Y) are also indicated by numbers (X/Y) above each panel. The blue dotted lines indicate the detection cut-offs for each assay. LRT, lower respiratory tract; ND, not detected; MN, microneutralization assay; URT, upper respiratory tract.

CDC tested 74 serum specimens collected <56 days after onset; 41 specimens were from 16 survivors, and 33 specimens were from 11 case-patients who died. Time between onset and collection did not differ between patient groups (survived, median 15 days, range 1–50 days; died, median 17 days, range 5–45 days). Four specimens from 4 survivors were collected ≈1 year after illness onset. Time courses of antibody responses are shown by patient in Figure 5 and Appendix 1 Figures 4–6.

Among case-patients who died, 5/6 had detectable neutralizing responses during the first 2 weeks of illness; by week 3, all of these case-patients with available specimens had detectable antibodies by MN (Table 4). Notably, 1 of these case-patients exhibited a 9-day delay in the development of a detectable response by VSV-MERS-S pseudoparticle assay compared with MN (Appendix 1 Figure 7); the detectable S-specific response by ELISA was also delayed for this patient. Overall, the 2 neutralizing assays were better correlated in specimens from survivors than from patients who died (Appendix 1 Figure 8).

**Table 4:** Specimens and MERS–CoV patients with detectable Abs, by time since illness onset and outcome, Saudi Arabia, August 1, 2015–August 31, 2016*

Outcome	Days since onset	No. specimens with Abs detected/no. tested					No. patients† with Abs detected/no. tested			
		MN	VSV-MERS-S	S ELISA	N ELISA		MN	VSV-MERS-S	S ELISA	N ELISA
Survived	<14	3/17	4/16	5/17	7/17		3/11	4/10	4/11	5/11
	14–20	4/8	4/8	4/8	6/8		3/5	3/5	3/5	4/5
	21–27	6/6	5/5	6/6	6/6		3/3	3/3	3/3	3/3
	28–55	10/10	10/10	10/10	10/10		4/4	4/4	4/4	4/4
	1 y	4/4	4/4	4/4	2/4		4/4	4/4	4/4	2/4
Died	<14	9/11	8/11	5/11	8/11		5/6	5/6	3/6	4/6
	14–20	9/9	6/8	7/9	9/9		4/4	2/3	4/4	4/4
	21–27	9/9	8/9	8/9	9/9		4/4	4/4	4/4	4/4
	28–55	4/4	4/4	4/4	4/4		3/3	3/3	3/3	3/3
	1 y	NA	NA	NA	NA		NA	NA	NA	NA

*Abs, antibodies; MERS-CoV, Middle East respiratory syndrome coronavirus; MN, microneutralization assay; N ELISA, nucleocapsid ELISA; S ELISA, spike ELISA; VSV-MERS-S, pseudoparticle neutralization assay; NA, not available. †Number of patients represents those with available specimens for a given period. Specimens were not available from all patients during all periods. Some specimens were not tested by VSV-MERS-S pseudoparticle assay because of insufficient volume.

Survivors and case-patients who died had responses by N- and S-ELISA (Table 4), although detectable N-specific responses preceded S-specific responses in 3 patients. By 1 year, N-specific responses had waned in 2 of the 4 patients tested.

### Co-detection of Antibodies and Viral RNA

We next compared neutralizing antibody titers to estimated viral load in the same serum specimen and to estimated viral load in respiratory specimens collected on the same day (Figure 6; Appendix 1 Figure 9). Among specimens with detectable antibodies by MN (Figure 6), viral RNA was often co-detected in serum, URT, and LRT specimens, even beyond 21 days after onset, when antibody titers were typically higher. Co-detection in serum (p = 0.032) and URT (p = 0.003) specimens was observed more frequently among patients who died than among those who survived.

## Discussion

We characterized MERS-CoV infection dynamics by patient demographics, underlying medical conditions, exposure route, and symptom progression and antibody responses by clinical outcome. Our findings demonstrate a possible association between DM and prolonged MERS-CoV RNA detection in survivors, when adjusting for severity. The prevalence of DM is high in Saudi Arabia; estimated national prevalence ranges from 14.4% (*41*) to 18.6% (*42*). DM is frequently reported among MERS case-patients (*7*,*43*), is a risk factor for illness among those with primary exposures (*13*), and has been associated with increased severity (*44*) and mortality (*26*,*44*), as was observed in our study. DM was less frequently reported in MERS patients from South Korea (*5*,*45*–*47*) in 2015, and its association with increased severity in that setting was less clear (*5*,*45*). In our investigation, factors affecting DM management before infection were unknown.

Information about MERS-CoV detection and antibody responses in case-patients who died has been limited (*19*,*24*–*26*,*29*). In our study, patients who died had robust neutralizing antibody responses during the second and third weeks of illness, but this response was not sufficient for patient recovery. During this same period (weeks 2–3), RNA levels peaked in the LRT of these case-patients, suggesting that antibodies might not be sufficient for virus clearance. Antibodies were more often co-detected with viral RNA in the serum and URT of case-patients who died compared with survivors. Co-detection of antibodies and RNA has been described previously but not by patient outcome (*19*,*25*). Six patients who died had MERS-CoV RNA in their serum, despite the presence of neutralizing antibodies, and 3 had RNA in urine. Detection of MERS-CoV in blood or serum has previously been associated with need for supplemental oxygen (*18*), the need for mechanical ventilation (*24*), and death (*24*,*26*). Although we detected RNA in the blood or serum of all severity groups, estimated viral loads might have been higher in patients who died than survivors; this was difficult to assess statistically because of limited specimen collection and variability in timing of collection. In 4/12 patients who died, RNA levels in the LRT decreased to low or undetectable before death, suggesting that viral replication in the respiratory tract might variably or indirectly contribute to outcome.

Previous studies using MN tests (*19*) or S-specific assays (*25*,*31*) have suggested that some case-patients who die might exhibit a delayed MERS-CoV–specific antibody response. Although most patients in our study developed early and concomitant MN and VSV-MERS-S (pseudoparticle assays) responses, 1 case-patient who died exhibited a prominent delay in detectable VSV-MERS-S and S ELISA responses compared with MN. This finding warrants further investigation and might suggest that the MN assay targets antibodies functioning beyond S-specific viral entry. Although our neutralizing assays targeted 2 different MERS-CoV strains, no variations exist within the receptor binding sites of these viruses (Appendix 1 Figure 10). Compared with these 2 viruses, the virus strain used in our S ELISA (EMC) differs in S by 2–3 aa, which is unlikely to confer a notable difference in binding during a polyclonal antibody response.

We further characterized virus shedding among mildly ill patients (i.e., those who did not require supplemental oxygen while hospitalized and who might typically be isolated at home) and found that RNA levels in the URT peaked during the first week of illness among this group, not in the second week as previously suggested (*18*), although LRT specimens were not available for included patients. Similar to previous descriptions, we detected MERS-CoV RNA in the blood or serum of some mildly ill patients (*18*,*25*), but we also detected RNA in stool up to 15 days after illness onset; viable virus was not isolated from these specimen types. MERS-CoV RNA has been reported in stool previously (*19*), but the severity of illness and the time since illness onset in these patients were unknown. Replication in the intestinal tract has been postulated (*27*), but its role in pathogenesis or transmission remains unclear.

We characterized symptom progression in mildly symptomatic patients and found that fever and cough (when present) typically aligned with MERS-CoV detection. However, some patients remained febrile or reported cough even after virus clearance from the URT. Given the variability we observed in symptom progression during the MERS-CoV detection period, testing for viral shedding should continue to inform patient management, as stated in current World Health Organization guidance (*14*).

Our investigation has several limitations. First, the number of patients enrolled might have been insufficient to detect some associations, especially when adjusting for other variables. Second, testing data were not available before onset for all but 1 patients, and so we used days from onset to MERS-CoV negativity to assess shedding duration; we also excluded fatal cases from such analyses because time to death was not reflective of shedding duration, meaning that factors associated predominantly with mortality were not assessed for prolonged shedding. Third, the presence of MERS-CoV RNA does not necessarily represent viable virus. Fourth, serum specimen collection was deemed too sparse to reliably assess antibody kinetics at the patient level.

Prolonged shedding in those with DM and the detection of MERS-CoV RNA from nonrespiratory specimens, including RNA-positive stool in mildly ill patients, should be considered in infection prevention and control and when determining whether home isolation is appropriate. The presence of detectable antibodies in case-patients who died and the co-detection of antibodies with viral RNA might also have implications for the development of vaccines and antibody therapeutics. Our findings broaden our understanding of MERS-CoV natural history and provide evidence to inform surveillance strategies, diagnostics, therapeutic and vaccine development, and clinical and public health management guidelines for MERS patients.

Appendix 1 Methods and additional information regarding Middle East respiratory syndrome coronavirus infection dynamics and antibody responses among clinically diverse patients, Saudi Arabia.

Appendix 2 Additional tabular information regarding Middle East respiratory syndrome coronavirus infection dynamics and antibody responses among clinically diverse patients, Saudi Arabia.
