# Supplementary material for: Middle East Respiratory Syndrome Coronavirus Infection Dynamics and Antibody Responses among Clinically Diverse Patients, Saudi Arabia
Source: Emerg Infect Dis. 2019 Apr;25(4):753–66. doi: 10.3201/eid2504.181595 (PMC6433025; doi:10.3201/eid2504.181595)
Supplement: Appendix 1 — Methods and additional information regarding Middle East respiratory syndrome coronavirus infection dynamics and antibody responses among clinically diverse patients, Saudi Arabia. [file 18-1595-Techapp-s1.pdf]

Article DOI: https://doi.org/10.3201/eid2504.181595

Middle East Respiratory Syndrome Coronavirus Infection Dynamics and Antibody Responses among Clinically Diverse Patients, Saudi Arabia

Appendix 1

Supplementary Methods

Serologic Assays

Serum specimens with sufficient volume were tested by using 4 CDC serologic assays: 1) microneutralization (MNt) assay using live Hu/Jordan_N3_2012 MERS-CoV (GenBank accession no. KJ614529.1) (*1*); 2) pseudoparticle neutralization assay (VSV-MERS-S) using vesicular stomatitis virus (VSV) expressing spike (S) domains 1 and 2 of Hu/Florida/USA-2_Saudi Arabia_2014 MERS-CoV (GenBank accession no. KJ829365.1) (see additional details below); 3) S ELISA (Ig-specific) using baculovirus-expressed domains S1 and S2 (ectodomain) of Hu/EMC/2012 MERS-CoV (GenBank accession no AFS88936.1) (*2*); 4) nucleocapsid (N) ELISA (Ig-specific) using bacterially-expressed N of Hu/EMC/2012 MERS-CoV (GenBank accession no AFS88936.1) (*1,2*).

Details of the Pseudoparticle Neutralization Assay (VSV-MERS-S)

Cells

BHK-21 (Baby Hamster Kidney-21) cells were cultured and maintained in Dulbecco’s Modified Essential Medium (DMEM) containing 5% fetal bovine serum (FBS) (Life Technologies/ GIBCO), and 1X penicillin/streptomycin (p/s) (Sigma) at 37°C under a 5% $CO2$ atmosphere. Vero (African green monkey kidney epithelial) cells were cultured and maintained in Dulbecco’s Modified Essential Medium (DMEM) containing 10% fetal bovine serum (FBS) (Life Technologies/ GIBCO), and 1X penicillin/streptomycin (p/s) (Sigma) at 37°C under a 5% $CO2$ atmosphere. Where production of different VSV based pseudotypes bearing MERS-CoV-S

and VSV-G glycoproteins were performed in BHK-21 cells, the neutralization assay and pseudotype particles titration were carried out in Vero cells. VSV pseudotype system components and BHK-21 cells were kindly provided by Dr. Michael Whitt.

Serum Samples

Pooled normal human serum (pNHS) was purchased from Lee Biosolutions and used as negative control serum. Human serum from a single patient with laboratory confirmed MERS-CoV infection was used as the positive control serum. The positive control serum was collected from the first imported case of MERS-CoV into the United States during the case investigation, with a neutralizing titer of 320. A laboratory confirmed SARS-CoV patient serum sample and a panel of human serum with confirmed high neutralizing antibody titers to human coronaviruses 229E, HKU1, OC43, NL63 were used in this study to evaluate VSV-MERS-CoV-S particle based neutralization assay for potential cross neutralization. A total of 52 human sera samples from MERS-CoV-infected patients in Saudi Arabia were used to examine equivalencies.

Production of Pseudotyped Viruses

A codon-optimized S gene was designed according to published MERS-CoV genome sequence Florida strain (GenBank accession number: KJ829365.1), synthesized by GeneScript and sub-cloned into pCAGGS 2.0 eukaryotic expression vector to construct pCAGGS-MERS-FL-S. Generation of VSV-MERS-CoV-S pseudovirus particles was performed as previously described (*3*). Briefly, BHK-21 cells were transfected with pCAGGS plasmid encoding MERS-CoV-FL-S (pCAGGS-MERS-FL-S), VSV-G (pCAGGS-VSV-G), or pCAGGS-empty vector (pCAGGS-EV) with Lipofectamine 2000 (Life Technologies). At 24 hours post-transfection, cells were infected with pVSVΔG*-G-Luciferase at a multiplicity of infection (MOI) of 5. Supernatants containing pseudoparticles were harvested 24 hours later and aliquots were stored at –80°C.

Titration of VSV-MERS-CoV-S Pseudovirus Particles

Pseudovirus particles titers were determined in Vero cells. Vero cells were infected in serum free Dulbecco's Modified Essential Medium (DMEM) (Life Technologies/ GIBCO) containing 1X penicillin/streptomycin (p/s) (Sigma), in triplicate wells of 96-well black and white (B&W) tissue culture treated plates (Perkin-Elmer) with 50 µL of 5-fold serial dilutions of

pseudoparticles. After 1 hr adsorption, the inoculums were removed, replaced with fresh DMEM containing 10% FBS (Life Technologies/ GIBCO), and 1X (p/s) (Sigma) media, and incubated at 37°C. At 24 hr post inoculation, luciferase activity was determined using the Luciferase Assay Kit (Promega, Inc) according to the manufacturer's instructions. The titer of VSV-MERS-CoV-S pseudovirus was defined as the highest dilution which gave rise to 105 relative luciferase units. For titration of VSV-MERS-CoV-S pseudovirus particles, the pCAGGS plasmids encoding vesicular stomatitis virus recombinant G and the empty vector (EV) pCAGGS-EV was used to generate VSV-G pseudovirus particles and pseudovirus particles without heterologous protein (EV) as positive and negative controls respectively.

Pseudotype Neutralization Assay

Briefly, Vero cells were seeded in 96–well black and white (B&W) tissue culture treated plates (Perkin-Elmer) 1 day before infection. Human sera samples were heat inactivated at 56°C for 30min, and diluted as 2-fold serial dilutions beginning with an initial dilution of 1:40 in serum free Dulbecco's Modified Essential Medium (DMEM) (Life Technologies/ GIBCO) containing 1X penicillin/streptomycin (p/s) (Sigma). Two hundred μL of each serum dilution were mixed thoroughly with 105 (relative luciferase units) equivalent VSV-MERS-CoV-S pseudovirus particles diluted to 200 μL and incubated at 37°C under a 5% CO2 atmosphere for 1 hour. One hundred μL of the pseudovirus particle-serum mixtures were transferred onto Vero cell monolayers and were incubated at 37°C under a 5% CO2 atmosphere for 1 hour. After adsorption, 100 μl of Dulbecco's Modified Essential Medium (DMEM) containing 10% fetal bovine serum (FBS) (Life Technologies/ GIBCO), and 1X penicillin/streptomycin (p/s) (Sigma) was added and incubated at 37°C and 5% CO2 for 23 hrs. At 24 hours post-inoculation, luciferase activity was determined using the Luciferase Assay Kit (Promega, Inc) according to the manufacturer's instructions. This assay was performed in triplicate.

To calculate neutralization, luciferase activity from each serial diluted human sera sample were normalized to pNHS. Neutralization was calculated as: (Average RLU value of pNHS - Average RLU value with test serum/Average RLU value of pNHS) X 100. Endpoint reciprocal dilutions were set as the highest dilution of the test serum sample where an 80% decrease in RLU was observed. Serum samples that failed to show at least 80% decrease at the 1:40 initial

dilution were considered negative. Positive and negative control serum were included to validate the assay.

References

1. Al-Abdallat MM, Payne DC, Alqasrawi S, Rha B, Tohme RA, Abedi GR, et al.; Jordan MERS-CoV Investigation Team. Hospital-associated outbreak of Middle East respiratory syndrome coronavirus: a serologic, epidemiologic, and clinical description. Clin Infect Dis. 2014;59:1225–33. PubMed http://dx.doi.org/10.1093/cid/ciu359

2. Trivedi S, Miao C, Al-Abdallat MM, Haddadin A, Alqasrawi S, Iblan I, et al. Inclusion of MERS-spike protein ELISA in algorithm to determine serologic evidence of MERS-CoV infection. J Med Virol. 2018;90:367–71. PubMed http://dx.doi.org/10.1002/jmv.24948

3. Whitt MA. Generation of VSV pseudotypes using recombinant ΔG-VSV for studies on virus entry, identification of entry inhibitors, and immune responses to vaccines. J Virol Methods. 2010;169:365–74. PubMed http://dx.doi.org/10.1016/j.jviromet.2010.08.006

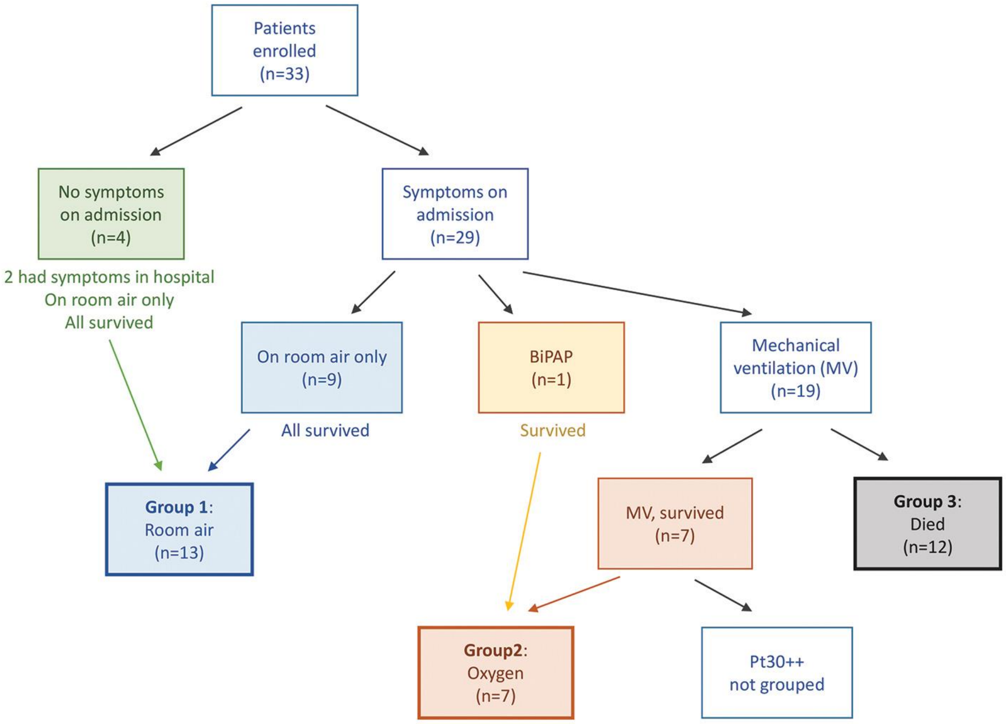

Appendix 1 Figure 1. Flow diagram depicting grouping of patients for severity analysis. ++, Pt30 was hospitalized and mechanically ventilated prior to MERS onset because of a road traffic accident; this patient was excluded from severity and clinical course analyses.

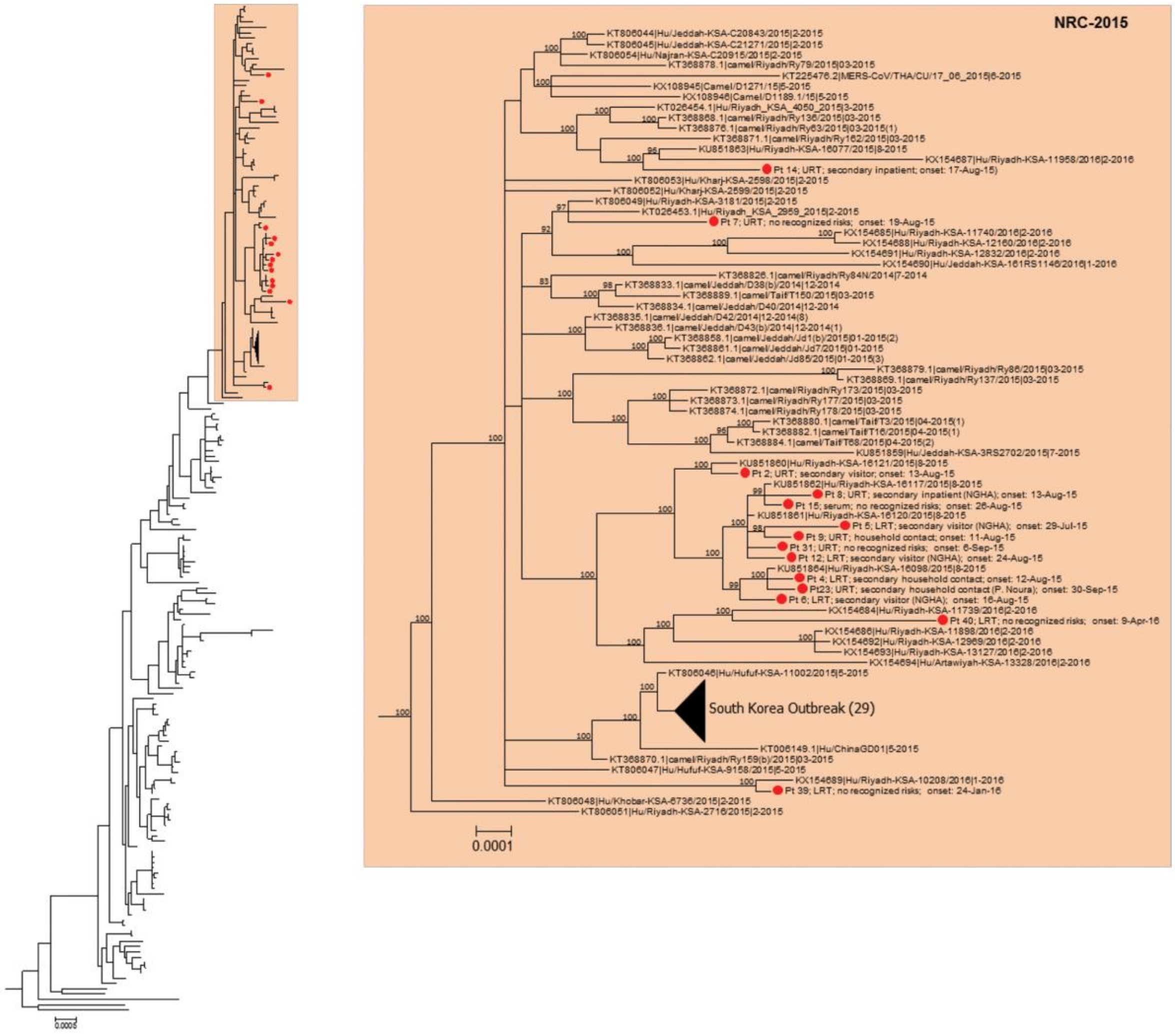

Appendix 1 Figure 2. Phylogeny of MERS-CoV full genome sequences. Phylogenetic analysis of the MERS-CoV genomes obtained from 14 patients in our study and 261 previously published sequences available in GenBank. The NRC-2015 (or lineage 5) subclade is repositioned for clarity. Whole genome sequences obtained in this study are marked with solid red circles. The tree was constructed by the program MrBayes v3.2.6 under a general time-reversible (GTR) model of nucleotide substitution with 4 categories of γ-distributed rate heterogeneity and a proportion of invariant sites (GTR+4 + I). Clade-credibility values ≥70% are indicated above the respective nodes. Numbers in brackets following some strain identifiers are the number of identical sequences with the same location and sample collection time. The scale bar shows the genetic distance as nucleotide substitutions per site. Each specimen label includes: accession number, city and country of collection, patient number (e.g. 040), day of collection post-enrollment (e.g. D1), specimen type (N, nasopharyngeal/oropharyngeal swab; S, sputum; P, serum), and date of collection.

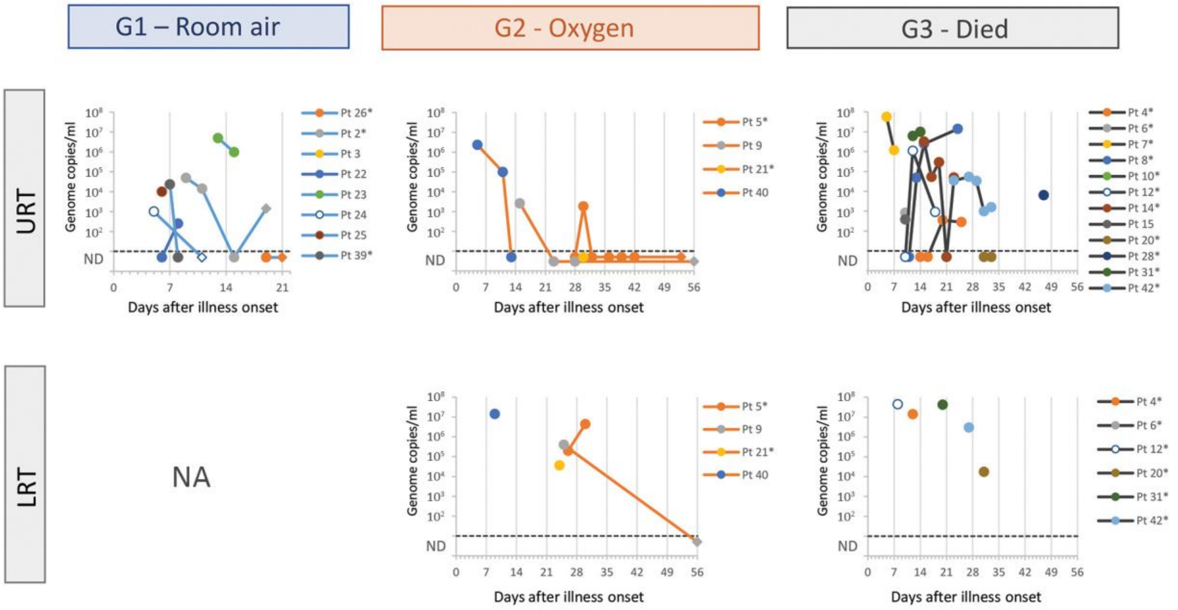

Appendix 1 Figure 3. Estimated viral loads in respiratory tract specimens collected from hospitalized patients and submitted to CDC. Upper respiratory tract (URT) and lower respiratory tract (LRT) specimens are shown by severity group. Estimated viral loads are based on upE rRT-PCR Ct values, or N2 Ct values if not available. The dashed line represents the limit of detection, below which specimens were considered MERS-CoV-negative or not detected (ND). *, patients with a documented history of diabetes mellitus (DM). Round data points represent specimens collected during the MERS-CoV detection period (defined by clinical results from respiratory specimens). Diamond data points represent specimens collected after the MERS-CoV detection period (defined by clinical diagnostic results from respiratory specimens); the URT specimen collected 19 days p.o. in Pt 2 is the only specimen with MERS-CoV RNA detected after the detection period in clinical specimens.

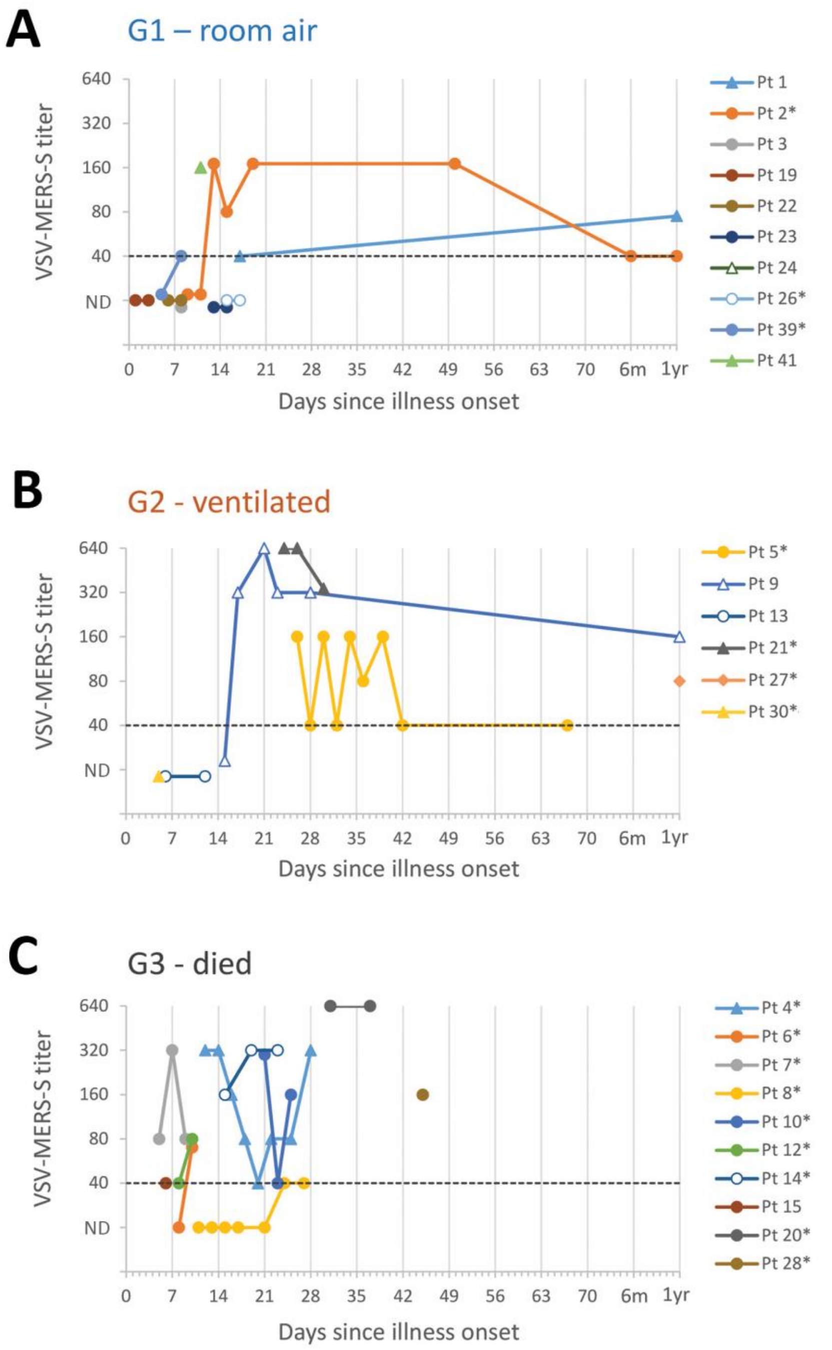

Appendix 1 Figure 4. Pseudoparticle neutralization (VSV-MERS-S) antibody titers, by patient and severity group. Panels A, B and C depict the VSV-MERS-S titers of sera collected from G1, G2 and G3 patients, respectively, by days since illness onset (day 0). The dashed line represents the limit of detection, below which specimens were considered not to have detectable antibodies (ND, not detected). *, patients with documented history of diabetes mellitus (DM). +, Pt 11 did not report any symptoms throughout their illness. ++, Pt30 was hospitalized and mechanically ventilated prior to MERS onset because of a road traffic accident.

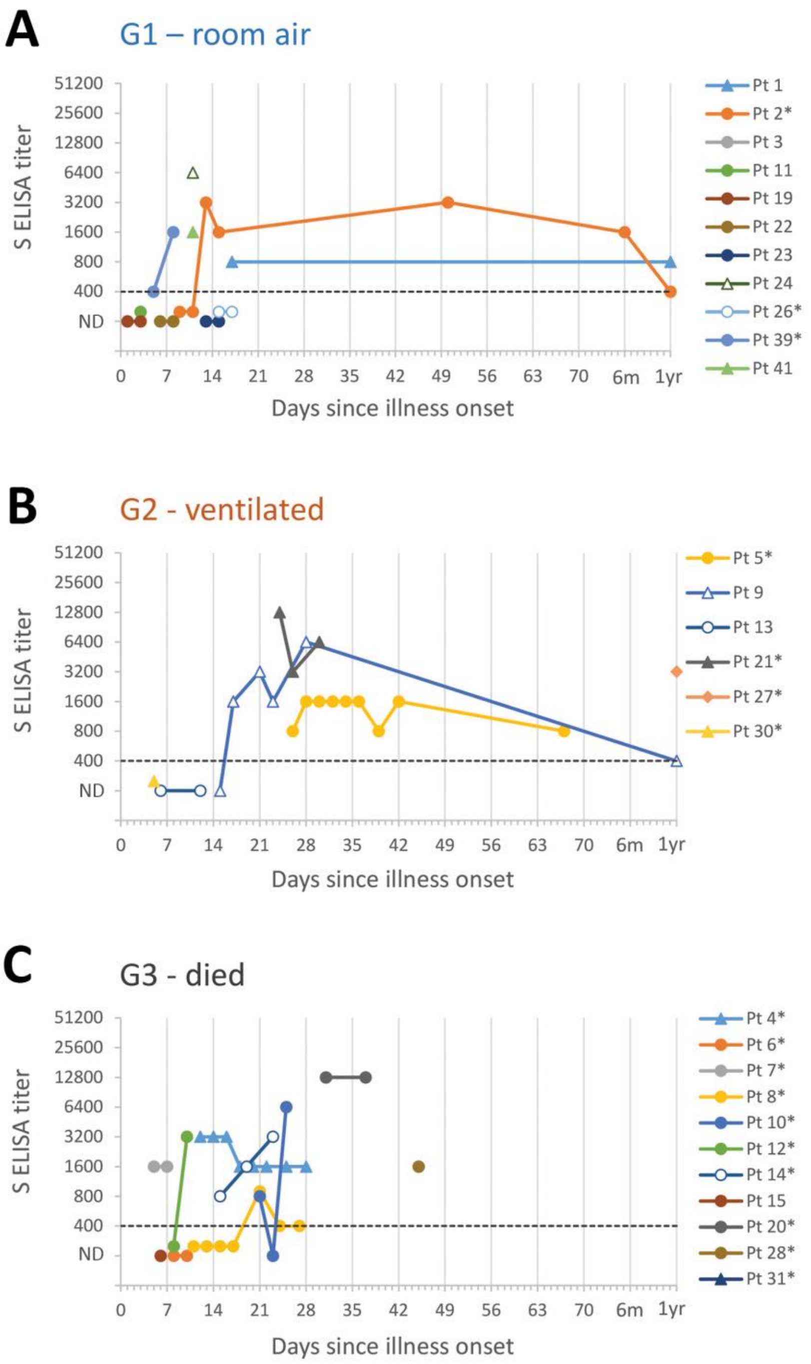

Appendix 1 Figure 5. Spike ELISA (S ELISA) antibody titers, by patient and severity group. Panels A, B and C depict the S ELISA titers of sera collected from G1, G2 and G3 patients, respectively, by days since illness onset (day 0). The dashed line represents the limit of detection, below which specimens were considered not to have detectable antibodies (ND, not detected). *, patients with documented history of diabetes mellitus (DM). +, Pt 11 did not report any symptoms throughout their illness. ++, Pt30 was hospitalized and mechanically ventilated prior to MERS onset because of a road traffic accident.

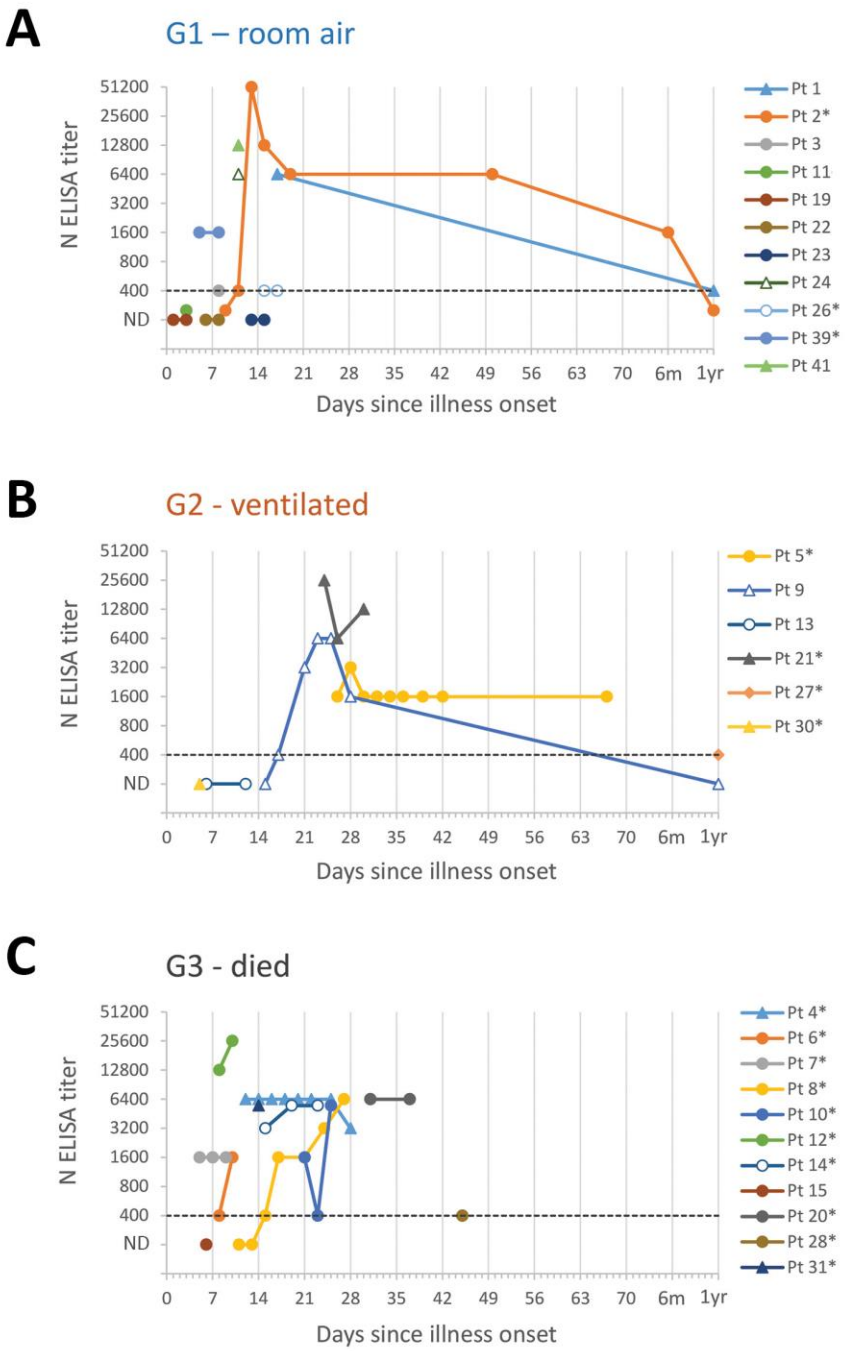

Appendix 1 Figure 6. Nucleocapsid ELISA (N ELISA) antibody titers, by patient and severity group. Panels A, B and C depict the N ELISA titers of sera collected from G1, G2 and G3 patients, respectively, by days since illness onset (day 0). The dashed line represents the limit of detection, below which specimens were considered not to have detectable antibodies (ND, not detected). *, patients with documented history of diabetes mellitus (DM). +, Pt 11 did not report any symptoms throughout their illness. ++, Pt30 was hospitalized and mechanically ventilated prior to MERS onset because of a road traffic accident.

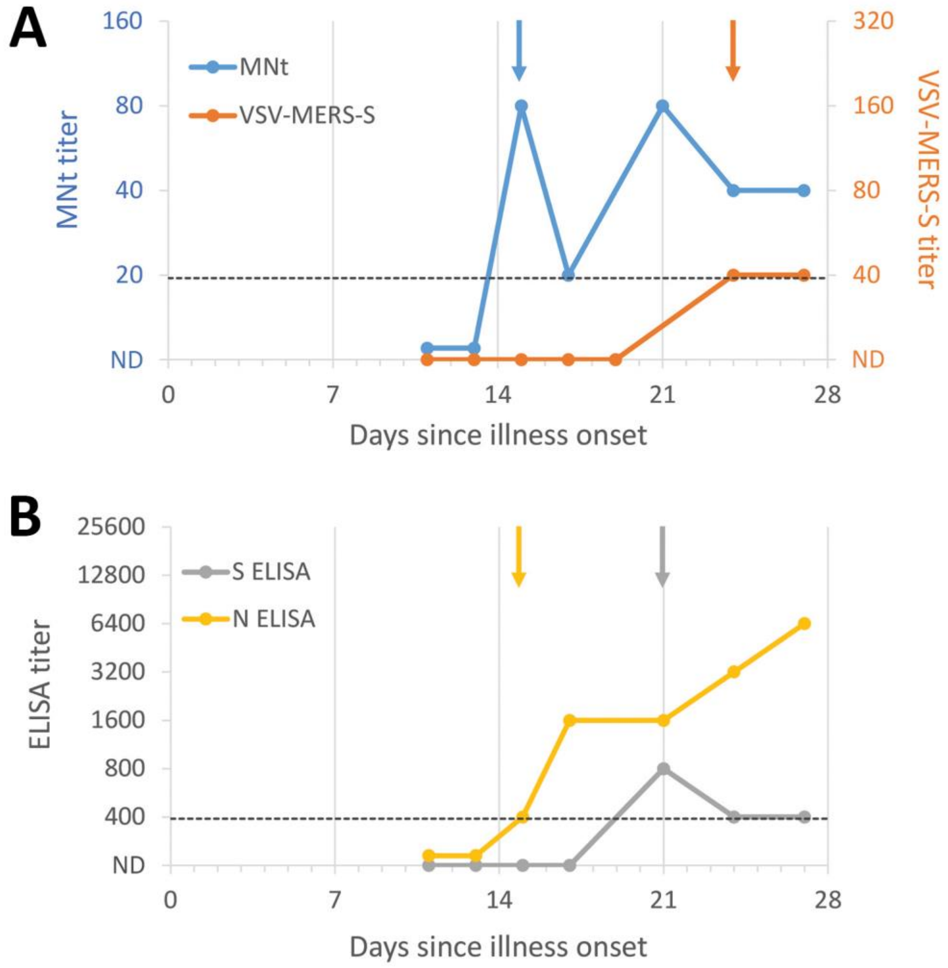

Appendix 1 Figure 7. Time course of antibody responses for Patient 8. Neutralizing (A) and ELISA (B) titers are depicted for patient 8, by days since illness onset (day 0). The dashed line represents the limit of detection, below which specimens were considered not to have detectable antibodies (ND, not detected). Arrows indicate the first day antibodies were detected in each assay.

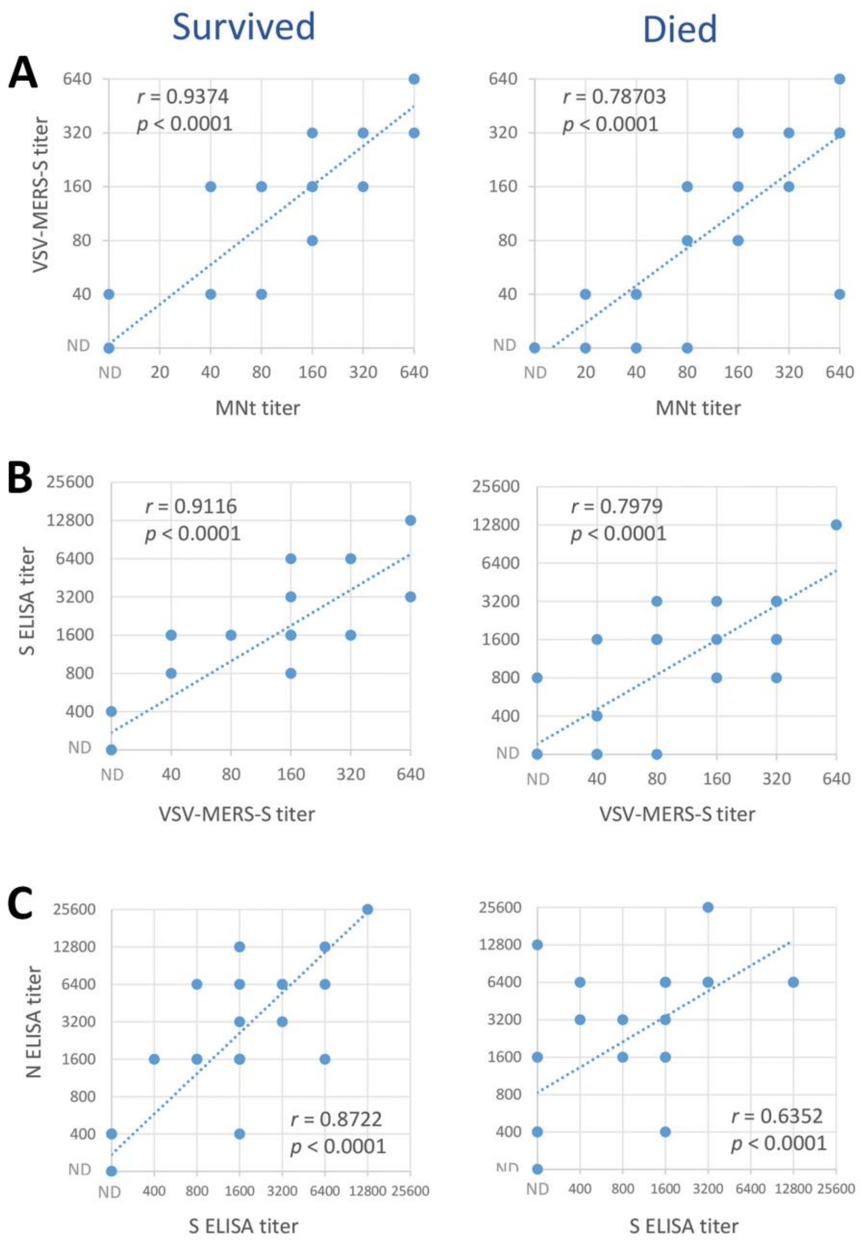

Appendix 1 Figure 8. Correlations of different serologic assays, by clinical outcome. Specimens collected <56 days post illness onset were included. Panel A depicts comparisons of microneutralization (MNt) and pseudoparticle neutralization (VSV-MERS-S) titers for each serum specimen. Panel B depicts comparisons of spike (S) ELISA and pseudoparticle neutralization (VSV-MERS-S) titers for each serum specimen. Panel C depicts comparisons of S ELISA and nucleocapsid (N) ELISA titers for each serum specimen. In each panel, specimens from patients who survived and died are depicted in the left and right graphs, respectively. Spearman r value and p values are depicted for each comparison.

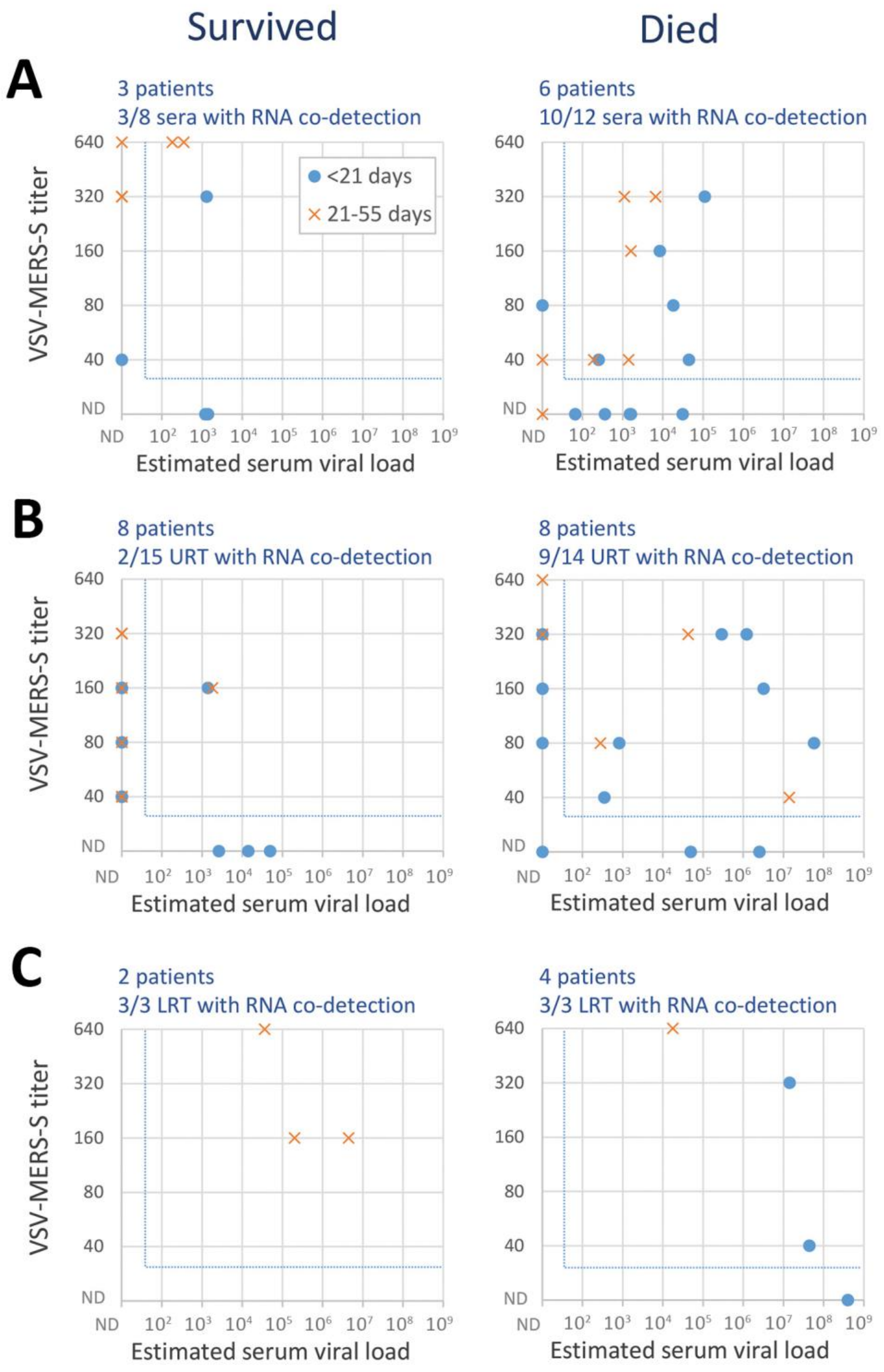

Appendix 1 Figure 9. Co-detection of pseudoparticle neutralizing serum Abs with RNA found in serum and the upper and lower respiratory tract, by clinical outcome. For each patient and specimen, pseudoparticle neutralization (VSV-MERS-S) titers of serum specimens were compared to estimated viral loads in the same serum specimen (A), or in upper (B) and lower (C) respiratory tract specimens collected on the same day from the same patient. We defined RNA co-detection as the detection of both RNA and

neutralizing Abs (VSV-MERS-S) in the same specimen, or in respiratory specimens collected on the same day from a given patient. We only included specimens from patients who were known to develop neutralizing Ab responses at any point during or after their illness. For the comparison in serum specimens, we only included specimens from patients who were known to have RNA detected in serum, at any point during their illness. For each panel, the number of patients included are indicated above the panel. The number of specimens with RNA co-detection (X) among those with detectable Abs (Y) are also indicated (X/Y) above each panel. The blue dotted lines indicate the detection cut-offs for each assay. ND, not detected. URT, upper respiratory tract. LRT, lower respiratory tract.

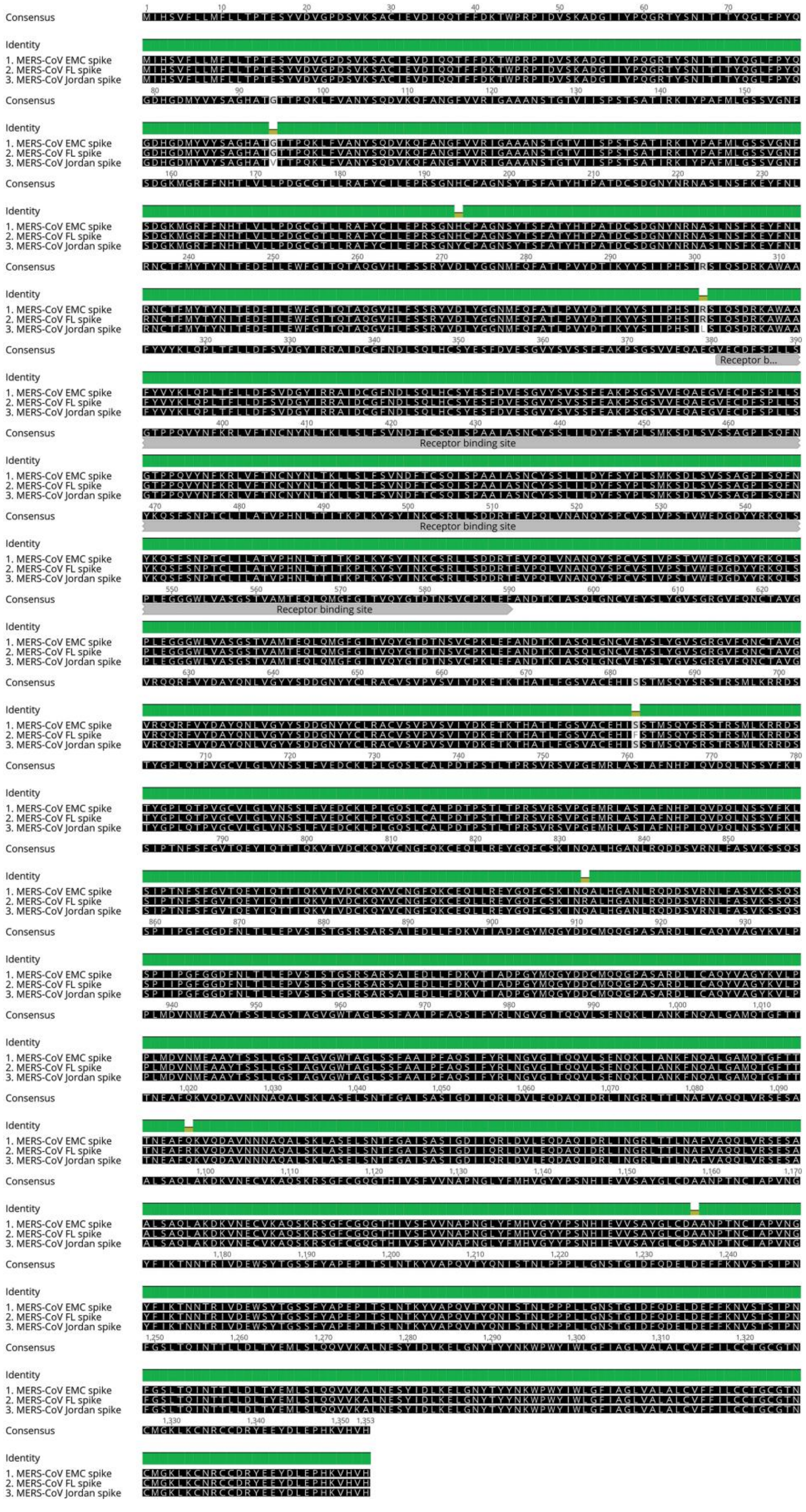
Consensus
Identity
1. MERS-CoV EMC spike
2. MERS-CoV FL spike
3. MERS-CoV Jordan spike
Receptor binding site

Appendix 1 Figure 10. MERS-CoV spike protein alignment of virus strains used in the serologic assays. MERS-CoV spike protein sequences from Hu/EMC/2012 (MERS-CoV EMC spike), Hu/Florida/USA-2_Saudi Arabia_2014 MERS-CoV (MERS-CoV FL spike), and Hu/Jordan_N3_2012 MERS-CoV (MERS-CoV Jordan spike) were aligned using Geneious (version 11.1.2) multiple sequence alignment tool. The alignment type used was global alignment with free end gaps with a Blosum62 cost matrix. Consensus sequence, identity, and the sequence of each spike strain are shown. Additional, residues within the receptor binding domain are annotated.
